# Consumer Choices and Service Quality in the University Canteens in Warsaw, Poland

**DOI:** 10.3390/ijerph16193699

**Published:** 2019-10-01

**Authors:** Ewa Czarniecka-Skubina, Hanna Górska-Warsewicz, Wacław Laskowski, Maria Jeznach

**Affiliations:** 1 Department of Food Gastronomy and Food Hygiene, Faculty of Human Nutrition and Consumer Sciences, Warsaw University of Life Sciences (WULS), 02-787 Warsaw, str. Nowoursynowska 166, Poland; 2 Department of Organization and Consumption Economics, Faculty of Human Nutrition and Consumer Sciences, Warsaw University of Life Sciences (WULS), 02-787 Warsaw, str. Nowoursynowska 166, Poland; hanna_gorska_warsewicz@sggw.pl (H.G.-W.); waclaw_laskowski@sggw.pl (W.L.); maria_jeznach@sggw.pl (M.J.)

**Keywords:** student canteens, quality, hygiene, customer service, consumer expectations

## Abstract

The purpose of this study was to identify and analyze consumer choices and service quality in university canteens in Warsaw. Our study consists of two parts. The first part of our research was conducted using a sample of 1250 adult respondents in 25 university canteens located at five higher education institutions. The reasons and frequency for using canteens, types of selected dishes and opinions on a given catering facility management system were analyzed. The second part of the study was conducted as an inspection to assess internal control and reliability of information. The respondents’ opinions are not in line with inspection assessments. This may be due to the fact that students do not pay attention to the quality of services in university canteens or have little knowledge about service, quality of services or hygiene aspects. For a detailed analysis of consumer choices and service quality assessment, we used Analysis of Variance (ANOVA) test and multi-dimensional cluster analysis. We identified four clusters regarding the type of meals and consumed frequency of consumption in university canteens, and five profiles in relation to evaluation of canteen interior, service and menu. In the correspondence analysis performed using the multidimensional Multiple Correspondence Analysis (MCA) method, we identified five clusters of consumers based on nine features, i.e., canteen location, frequency of using the canteen, gender of respondents, dwelling place, financial status of respondents. Our research on the functioning of university canteens is one of the first not only in Poland, but also in the countries of Central and Eastern Europe. The evaluation of the quality of nutrition in canteens should be continued in order to prevent diet-related diseases. Based on the results of our research, we postulate to introduce an evaluation guide for university canteens taking into account various aspects of services.

## 1. Introduction

Canteens play an important role in the daily nutrition of many people, including students. A well-balanced, satisfying and healthy diet is just as important to a busy university/college student as the correct class schedule, teachers or learning environment. The university canteen should propose menus that promote healthy diet, but at the same time not limiting their food choices, including new trends in nutrition. Young adulthood is a particularly important time for the promotion of healthy eating, because during this period, nutrition habits are developed and remain all through one’s lifetime [1]. For many student’s, going to the university is a transitional life stage which may include significant changes. They are leaving the family home, starting studies at university, meeting new people, friends and partners, as well as starting a part-time job. Some of them even become parents. Many people lack interest in following a healthy and balanced diet. This depends on their life, nutritional behavior and financial situation [2,3,4,5]. Apart from students, the university staff also use the canteens.

Service quality can be described briefly as a phenomenon considered within the context of customers’ expectations and perceptions about the service offered. According to El-Said and Fathy [6], the food service on campus is more complex, diverse and dynamic. Rendering the measurement of service quality and identifying of the determinants of service quality makes it difficult. The quality of services can be examined in different ways. These include Service Quality (SERVQUAL) or the Dining Service (DINESERV) model [7,8]. They can also be dedicated methods [9,10,11,12,13,14].

According to several authors [1,15,16,17,18,19,20,21,22,23,24,25], customers’ expectations and perception of service will determine the perceived quality of service. The customer satisfaction is a major determinant of a company’s long-term profitability, and customer loyalty [15]. There are different factors influencing the choice of canteens by students and u staff, the choice of food in canteens and the satisfaction of students and university staff from canteen services. Some of the factors are as follows:

- The quality of food and beverages, quality of ingredients used in foods, taste of food [1,16,17,18],
- Menu variation [16,19,20];
- Quality of service (staff performance and ambience), efficiency of service [1,16,18,21,22]; responsiveness-assurance, empathy-equity, reliability and tangibles [4];
- Value, price and price fairness [1,20];
- Hygiene and cleanliness, food hygiene and environment, cleanliness while serving to customers [20];
- Location, as well as the atmospherics of establishment [1,18,19], the canteen design [23], the physical setting [26], furniture suitably designed at university canteens [20];
- Waiting time [16].

According to some other authors, the perceptions of university food services tend to be unfavorable [27]. Various factors are responsible for this, such as the diverse situational, contextual, and environmental constraints, including repetitive consumption of limited and monotonous menu, low quality of food and service, as well as facility in general [27]. Other studies indicate that meals offered in canteens do not comply with nutritional recommendations, which affected on the low level of satisfaction from services [28,29]. Food safety of consumed meals is not fully guaranteed [22]. The hygienic aspects of food handlers and eating environment had significant and high impact on satisfaction of food service quality according to some authors [2,22,30]. A major risk of food contamination is associated with the food handlers [2]. 

Various aspects of customer services in university canteens are also important. The unsatisfactory services offered by the university cafeteria operators, staff unfriendliness, unresponsiveness (lack of smiles and greetings) slow service and unreasonable product prices was stated by Chang et al. [20]. Service quality was found to have a significant effect on customer satisfaction [4]. According to De Silva [31], the overall service quality contains three major variables; cleanliness of the canteens, employee commitment as well as physical layout of the canteens. Edwards [32] stated that when examining eating out, it is important to consider not only the food or meal, but also the customer and the situation under which the consumption will take place.

So far, there are a few publications on the quality of services in university canteens [28,33,34,35]. A few of them contain different aspects of the quality of nutrition in university canteens [28,35], ensuring proper hygiene conditions for food production [33] and customer service [36]. Research results focused mainly on pre-school and school canteens [28,36,37,38,39,40,41,42]. 

A study in a Belgian university canteen [28] showed a negative association between canteen usage and healthy habits when the nutritional quality of the meals were low (high fat and fruit are available only for a surcharge). They also showed that meals in the university canteen lacked fruit and vegetables and included too much salt and fat [28]. According to Guagliardo et al. [43], eating regularly at university canteens was less frequent among less well-off students and was positively associated with some healthier self-reported dietary habits. Moreover, a study by Tacken [44] indicates that most students do not consider their eating patterns important and making healthy food choices is not a top-of-mind issue for them. It is known that the university cafeteria and overall food environment decreases students’ intention to make healthy food choices due to the plethora of less healthy foods they must navigate through at every meal opportunity [44]. The results of our research may become a postulate for those planning menus to provide a menu that is more desirable from a nutritional point of view. 

Meals and beverages are consumed during breaks, which are social events for most students whereby they communicate and hang out with each other. It is very likely that most students’ choices on what to eat are largely based on impulsive decision. Probably, students, when they are at the canteen deciding what to eat for lunch, will most probably not be motivated enough or too distracted to engage in deliberate decision making about their eating behavior. Chang et al. [20] stated that students’ satisfaction with the university cafeteria is influenced more by food quality than staff, price fairness and ambience. 

The nutrition of adults in canteens seem equally important. University staff and studying young adults spend a lot of time at the university in various types of classes. Often times, even 12 h per day. Contemporary students after graduation, will become customers of catering establishments in various enterprises, offices, etc. Developing the habit of eating a meal while away from home can be one of the key conditions for maintaining good health, satisfaction with life and work. Nowadays, the quality of gastronomic services, also in canteens, should be considered in a broader context. Only in those canteens where the quality of services is at a high level in terms of the health quality of meals, hygienic quality of food production and consumer service, can an appropriate nutritional and hygienic behavior be formed. 

In Europe, most of the research pertain to canteens in primary schools, colleges, but not universities [36,37,38,39,40,41,42,45]. It also concerns a different age group of consumers and canteen selection factors, not service evaluation. There is no comprehensive assessment, as in our study. Consumers change their eating habits, hence the need to assess university canteens, especially since this subject has not been discussed in Poland, and in Europe, such research has been scarce. In Poland, canteens at universities are run by private companies that take part in competitions for mass catering contracts. There is no government funding. Only customer interest and their satisfaction from food service allow the facility to remain on the market. However, the law of supply and demand does not quite operation here, because students have only a few minutes to eat meals, buildings of many Universities are usually located on the outskirts of the city. For this reason, they are often the only place where you can have a meal during your stay at the University. We analyze Polish canteens due to the fact that the number of foreign students studying in Poland on a continuous basis (3 and 5-year studies) as well as those coming here for various programs, including Erasmus, is increasing. 

Based on the above conditions, we have undertaken research aimed at identification and analysis of consumer choices and service quality in university canteens in Warsaw. To fulfill this goal, we have divided our study into two parts. The first part was conducted among consumers using university canteens, while the second part was an inspection analyzing various aspects of canteen activity. 

## 2. Materials and Methods 

### 2.1. Consumers’ Opinion about Canteens 

Our research was conducted using a sample of 1250 adults respondent in 25 canteens (also called cafeterias) at five higher education institutions (universities, polytechnic, academy, marked A,B,C,D,E) in Warsaw, Poland. The respondents completed the questionnaire in the presence of the interviewer, so that, they could ask an additional question at any time or make sure that the content of the question was properly understood. In each canteen, the opinion of 50 customers using the services of establishments, and who agreed to fill in questionnaire was examined. Analyses related to this paper were designed as a study with a convenience sampling. Research was carried out during the person’s stay in the canteen.

The questionnaire was designed by us, based on literature [7,8] and our previous research [9,10,11]. Questions for inspection was design base on our previous research [12,13,14]. The questionnaire was validated by means of a pilot study with 20 people. All problems have been identified, for example, unintelligible questions and questionnaire construction. The questionnaire has been completed and amended. The data was collected by well-trained interviewers. Each respondent who had agreed to take part in the study was invited to fill the questionnaire. Explanations were given if necessary. 

The questionnaire structure is presented in Table 1. Questionnaire consists of two parts. First part of the questionnaire consists of five questions relating to canteens. The questions pertained to reasons and frequency of visiting canteens, type and frequency of meals consumed, as well as evaluation of features of the given canteens. The assessment was based on canteen interior, customer service and canteens’ offer. The second part of the questionnaire was related to respondent’s sociodemographic details. 

Characteristics of respondents are presented in Table 2. The study involved mainly young educated women, with secondary school or bachelor/engineer degree, and university staff with different of dwelling places and financial status, which results from the selection of a sample among customers of canteens during meals. The respondents were free to participate in the research. 

### 2.2. Canteens Functioning Assessment

The second part of the study was conducted as an inspection to assess internal control, reliability of information, and expressing opinions on a given catering facility management system. It was carried out visually by a trained auditor in each of the 25 evaluated student canteens of universities in Warsaw, similar to the first stage of our research. The inspection questionnaire consisted of the following parts:

- A description of the canteens (number of consumer seats/tables, opening hours, number of operating personnel);
- Assortment (type of meals and beverages, differentiation of prices),
- Cleanliness and hygiene of rooms and tableware (cleanliness/hygiene in the canteen, cleanliness of tables, floor and tableware),
- Personal hygiene of staff (working clothes, the use of headgear, neatness of clothing, use of disposable gloves, separating money from food, hygiene handling with food);
- Customer service (welcome, farewell, speed of service and order processing time);
- Attractiveness of canteen (features such as: interior design, attractiveness; music—variety and volume, time spent waiting for order, sugar availability, availability of spices, availability of disposable napkins, promotion—discount),
- *Meals* (variety of meals and beverages, nutritional information in menu, vegetarian offer, healthy foods menu offer, the method of meals storage, allergens information).

The purpose of the inspection was to evaluate each canteen and to compare features such as: prices, variety of meals, hygiene levels, etc. between individual canteens. Individual aspects were assessed in the range of 0—unsatisfactory, 1—satisfactory. A total of 25 points was the maximal amount of points which could be obtained. 

### 2.3. Data Analysis

The statistical analysis of the results was performed using Statistica software (version 13.3 PL; StatSoft Inc., StatSoft, Krakow, Poland). The ANOVA test and multi-dimensional cluster analysis was used. Significance of differences between the values was determined at a significance level of *p* < 0.05. For a more comprehensive and full presentation of consumer behavior in the canteens, it was the Kohonen’s neural network was implemented [46]. The analysis allowed the classification of respondents by combining them into groups (clusters) on the principle of similarity of answers to many questions. The averaged responses for a given cluster constituted a response profile. These profiles were further analyzed while looking for affinities with similarly obtained profiles, but in relation to other blocks of questions or to variants of answers to simple questions. To identify affinity, correspondence analysis and correlation tables were used. When designing the network, linear architecture was adopted, i.e., neurons in one row, their number did not exceed 11, 100% of cases were always involved in the learning, the number of learning epochs was 400, the neighborhood was excluded, the learning rate varied from 0.5 to 0.1. 

The quality of the classification was assessed based on the correlation ratio and subjective judgment regarding the disproportion between the numbers of the largest and smallest clusters of cases. It was assumed that the cluster centroid presents its profile (model) of the multidimensional system. In separate exploratory classifications conducted by conversation, the different numbers of profiles were obtained. After their assessment, the classifications were finally accepted with:

- Four profiles of the subject of consumption (what it consumes?);
- Eight profiles of the frequency of consumption in the canteen;
- Five profiles for evaluation of canteen interior, service and menu.

The results of exploratory analysis were confronted, including simple questions, using correspondence analysis. Correspondence analysis was performed using the multidimensional Multiple Correspondence Analysis (MCA) method [47].

## 3. Results

### 3.1. Opinion of Consumers about University Canteens

#### 3.1.1. Frequency and Factors Determining the use of Canteen Services by Respondents

The frequency of university canteens use was independent of gender, age and dwelling place (*p* > 0.05). However, it depended on education and financial status (*p* = 0.0011). A significant percentage of respondents used the offer of university canteens sporadically (39.4%). Similarly, a large group of consumers (35.5%) used canteens systematically, at least once a week or daily. Others used canteens 2–3 times (18.6%) or once a month (6.5%). 

Among the reasons for using canteens, the respondents mentioned: meal’s consumption (73.6%) or other reasons: rest between lectures and classes (11.5%); social meeting (9.5%); preparation for classes (3.7%) and others (1.7%).

#### 3.1.2. Types of Meals and Frequency of Consumption by Respondents in University Canteens 

Respondents declared that they buy in the canteens: beverages (45.5%), soups (39.2%), sandwiches (28.0%), main course—full set (24.2%) or main course—incomplete set (13.2%). A relatively large percentage of respondents (24.5%) indicated purchases of fast food dishes (hamburgers, hot-dogs, French fries etc.). A smaller percentage of respondents (13.1%) pointed that they were buying salads. The least percentage of people were buying desserts—6.6% and fruits—2%. 

**Figure 1:**
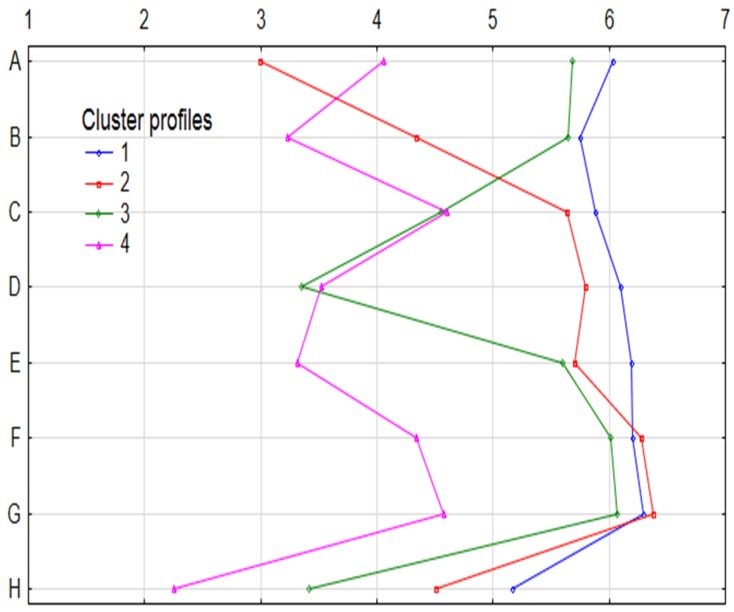
Profiles and frequency of using the canteen offer. 1—daily, 2—2 or 3 times a week, 3—once a week, 4—2 or 3 times a month, 5—once a month, 6—sporadically, 7—never; A. soups, B. main course, C. Fast food, D. sandwiches, E. salads, F. Desserts, G. Fruits, H. Beverages.

Among the consumers using University canteen services, based on the answers, 4 profiles were selected to summarize the behaviors of 4 consumer clusters - canteen usage profiles (Figure 1):

- They use it rarely, sometimes buying a beverage (Fr.1)– *n* = 609; 48.7%;
- They use it once a week when buying soup, less often the main course or beverage (Fr.2) – *n* = 321; 25.7%;
- They use it once a week, buying a sandwich or beverage (Fr.3)– *n* = 215; 17.2%;
- They use it systematically, buying various dishes (Fr.4)– *n* = 105; 8.4%.

As a result of statistical processing, multiple indications showed 10 models/patterns of consumer responses. Customer clusters (since Co-1 up to Co-10) explained what consumers most often eat in the canteen. Most often, respondents (Co-1, *n* = 202, 16.2%) went to the university canteens to eat a main course and usually soup (set), only beverages (Co-2, *n* = 183, 14.6%) or only soup (Co-3, *n* = 159, 12.7%). Quite a significant percentage of consumers bought: fast food meals (Co-4, *n* = 135, 10.8%), soup and beverage (Co-5, *n* = 130, 10.4%), a sandwich and beverage (Co-6, *n* = 125, 10%) or only a sandwich (Co-7, *n* = 120, 9.6%). The smallest percentage of consumers bought an incomplete main course (Co-8, *n* = 84, 6.7%), a set consisting of a soup, main course and beverage (Co-9, *n* = 62, 5%) or dessert with beverage (Co-10, *n* = 50, 4.2%). 

Analyzing the data, it can be stated that the significant number of consumers are characterized by mutually exclusive behaviors. This is demonstrated by weak, insignificant negative correlations. Examples included behaviors that the canteen consumers rarely choose other menu items, except soup, when choosing a main course (*r* = 0.44). When they are buying a sandwich, they do not buy a main course or soup. In this case, they rather settle for salad (*r* = 0.18). Other correlations indicate that the purchase of a fast food dish with a soup or main course was excluded. The purchase of a fast food type dish, however, was related to the purchase of a drink (*r* = 0.42), or the purchase of dessert (*r* = 0.66).

#### 3.1.3. Evaluation of Canteens by Respondents 

**Figure 2:**
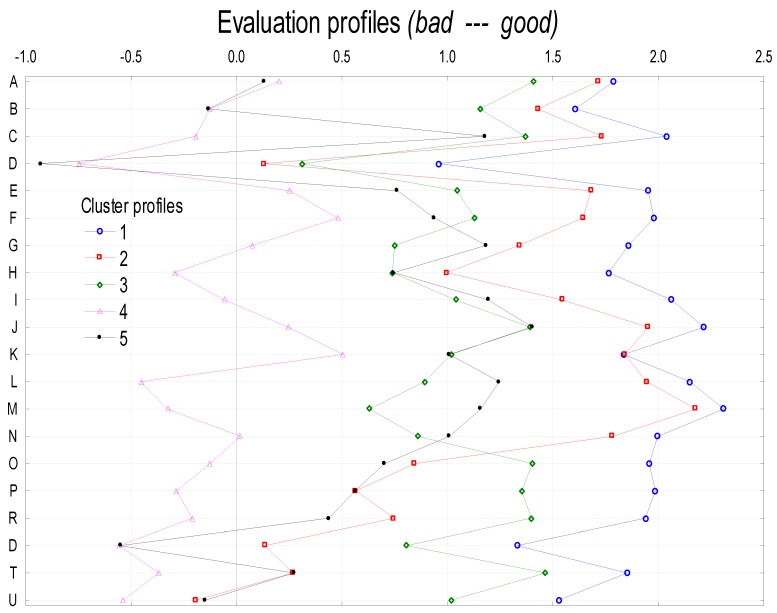
Profile of evaluation of service quality. Evaluation of canteen interior: A. interior design, B. equipment attractiveness, C. establishment atmosphere, D. music, E. table cleanliness, F. floor cleanliness, G. sugar availability, H. spice availability, I. disposable napkins availability f; Evaluation of customer service: J. speed of service (order processing time), K. service time (queuing), L. staff professionalism, M. staff politeness, N. staff outfit; Evaluation of canteens’ menu: O. quality of soups: taste, smell, the way of serving, P. quality of main course: taste, smell, consistency, the way of serving; R. variety of meals, S. availability of healthy meals, T. prices of soups, U. prices of main course; Evaluation scale: (−2) very bad; (−1) bad; (0) no opinion; (1) satisfactory; (2) good; (3) very good.

Five assessment profiles (clusters) of canteens were selected among the respondents (Figure 2). The first cluster (Eval. 1) found consumers (35%) who rated the functioning of canteens highly. The second cluster (Eval. 2) found people (22.8%) who rated canteens services highly but were critical of quality and prices. The third cluster (Eval. 3) included people (14.1%) who rated the functioning of the canteens high but were critical in many areas. The fourth cluster (Eval. 4) included people (9.5%) assessing the functioning of canteens critically. In the last cluster (Eval. 5) there were insightful and ambivalent people (9.1%). 

**Figure 3:**
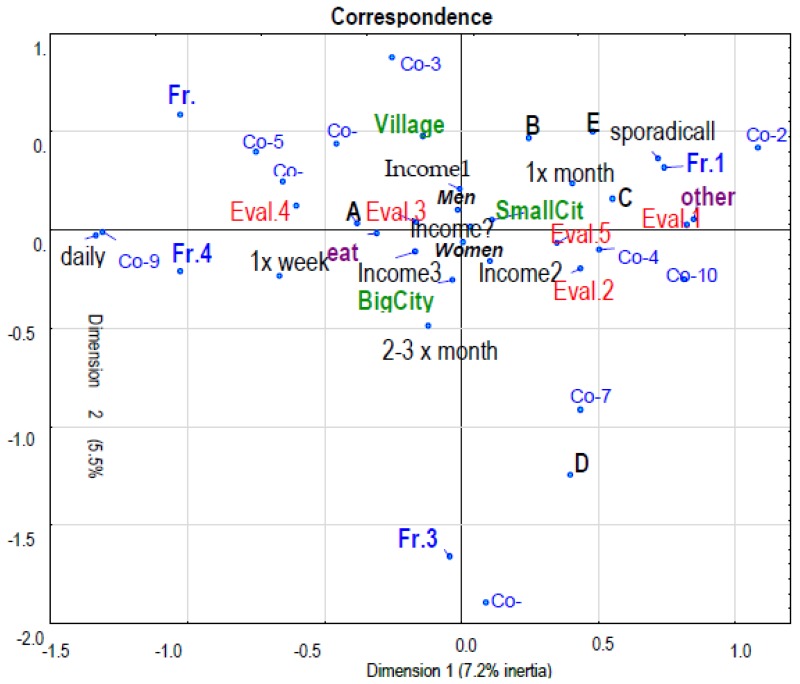
Correspondence graph of individual variants of respondents’ answers. University: A, B, C, D, E; Gender: Men, Women; Dwelling place: Village, Small City (City up to 100,000 inhabitants), Big City (City over 500,000 inhabitants); Financial status: Income 1—low, Income 2—average, Income 3—high, Income?—not given; Reasons: eat—meal’s consumption; other reasons—rest between lectures and classes, social meetings, preparing for classes, other; Profiles of general frequency of using the canteen: sporadically, once a month, 2–3 times a month, once a week, daily; Profiles of consumption: Co-1, Co-2, Co-3, Co-4, Co-5, Co-6, Co-7, Co-8, Co-9, Co-10; Profiles of frequency of use 8 types of meals: Fr.1, Fr.2, Fr.3, Fr.4; Profile of Evaluation: Eval. 1, Eval. 2, Eval. 3, Eval. 4, Eval. 5.

The correspondence analysis was performed using the multidimensional MCA method. After the experimental stage, the following features were finally considered; there were different numbers of variants:

(1) Canteen location, five variants (A,B,C,D,E),
(2) General frequency of using the canteen, five variants (sporadically, once a month, 2–3 times a month, once a week, daily);
(3) The reason for using the canteen, two variants (eat, other),
(4) Gender of respondents, two variants (men, women),
(5) Dwelling place, three variants (village, small city, big city),
(6) Financial status of respondents, four variants (low, average, high, not given),
(7) Profiles of consumption from previous own exploratory analysis, 10 variants (Co-1… Co-10),
(8) Profiles of frequency of use taking into consideration eight types of meals, four variants (Fr. 1, Fr. 2, Fr. 3, Fr. 4),
(9) Profiles of evaluation of canteens from previous own exploratory analysis, five variants (Eval. 1, Eval. 2, Eval. 3, Eval. 4, Eval. 5), Figure 3.

The correspondence graph (Figure 3) showed affinities and co-occurrence by deploying a total of 40 variants from nine features in a two-axis coordinate system, in which the abscissa represented the first factor of the analysis, and the ordinate—the second factor in order of importance. Both factors explained almost 23% of the total system inertia. The scale of the explanation is not large, there are other systems of conditions (factors), which means that shaping consumer motivation is indeed complex and probably highly individualized. 

The first and the second factor are quite clearly related to the variants of the feature “What is consumed”. On the horizontal axis there are spaced variants symbolically marked; Co-9 located on the left, and the Co-2 option located furthest on the right. These variants mean: the first is the one involving the order of a full set, i.e. soup, main course and a drink and the other is just a drink. The other variants of this feature are distributed between these variants. Closer to the Co-2 (beverage) variant is Co-5 (soup + beverage), Co-8 (incomplete main course) and Co-1 (main course with eventually soups), while on the other side of the axis closer to the Co-2 (beverage) variant is Co-10 (dessert).

In summary, we have a system from consuming a meal to satisfying thirst or “whims” (dessert). This arrangement includes the distribution of variants of the feature “How often it uses”. As follows, from the left side, i.e. the side of the meal, we have variants daily, and then once a week and then seeing to the right, i.e. in the direction of dessert and drink, several times in the month and the most right - “sporadically”. Therefore, we notice the rule, that more frequent use is accompanied by a more complete nutrition (full set), and less frequent use goes beyond satisfying the desire or consumption of dessert, possibly resulting from a specific circumstance, which is also indicated by the location in this area for another reason to visit the canteen rather than consume a meal.

This area also includes the variant of the feature “How often you eat individual types of meals”, namely the Frequent-1 variant, which means the response profile that mainly uses the response “sporadically” for all meals or products listed in the survey, except for beverages, where in this profile “once a month” dominates. It is worth noting, that the meal side of the first factor is accompanied by a rather restrained evaluation, while the drink and dessert side is perceived as being more positive. Consumers of canteen A expressed a more reserved assessment. Income and dwelling place proved to be less significant. 

The second factor indicates the affinity of consumers in canteen D to purchase sandwiches more often, possibly in the variant extended to include a drink, the variant Frequency-3 also corresponds to this. A negative affinity for this constellation of variants have the variant of the feature “What consume” with the answer: “Soup”.

Our consumer typography includes five clusters. People from Cluster 1 (*n* = 484) evaluating university canteens highly have been called Enthusiastic. In Cluster 2 (*n* = 315) there is people who rate university canteens highly, but criticize the quality of service and the prices of dishes; we called them Medium-Enthusiastic. Cluster 3 (*n* = 194) included people who rated university canteens highly, but criticized many aspects of their quality; we called them Medium-Critical. Cluster 4 (*n* = 131) is people who critically evaluate university canteens; we called them Critical. Cluster 5 (*n* = 126) included people carefully assessing university canteens but ambivalently; we called them Ambivalent. These groups were statistically different in terms of gender (*p* = 0.03211), age (*p* = 0.01076) and financial status estimated in their own opinion (*p* = 0.00895), but did not differ statistically in terms of education (*p* = 0.44691) and dwelling place (*p* = 0.82528). 

### 3.2. The Results of the Inspections of the Quality of Services in University Canteens

All evaluated university canteens offered their services for 8 hours a day. The estimated canteens varied in number of customer places (23–100, median 58) and tables (6–36, median 14). There were one to three operational staff (median two) available to serve customers. The staff had 16 to 64 clients at one time (median 33) to service.

The rated canteens differed in terms of aspects assessed. Inspection results indicate low notes for the parameters assessed. They were rated at 17 points (median) in total, which according to initial assumptions, shows that the quality of services was at an unsatisfactory level. While the hygiene was estimated at about 5 points (median), and consumer service at 4 points (median). Meals were rated the highest—6 points (median). In all evaluated university canteens, various factors that reduce the quality of services were found (Table 3).

Hygienic aspects. The assessment covered the hygiene of the dining area and tableware, as well as personal hygiene of the staff (Table 3). Unsatisfactory cleanliness of tables in 5 canteens, as well as unsatisfactory cleanliness of floors in six canteens was found. 

On the other hand, the hygiene of the tableware (plates, cutlery, glass) was assessed as satisfactory. In personal hygiene of staff, more irregularities were found. They concerned mainly, the lack of protective clothing and wearing a headgear (in 14 canteens), the neatness of the staff (in 9 canteens), as well as not using disposable gloves, and failure to separate money from food.

However, the handling of food during its production and distribution was found to be satisfactory.

Consumer services aspects. The inspection revealed many irregularities in the area of consumer service. The low quality of customer service is correlated with number of staff (*r* = −0.44, *p* < 0.05). Most canteens did not implement the basic activities of the staff towards the client: greeting and farewell (in 20 and 22 canteens, respectively). The speed of service (in eight canteens) and the time of order completion (in six canteens) was unsatisfactory too. This is due to the fairly large number of clients to be served by one staff. Only in one1 canteen the number of clients to serve was 16 by one staff employee, in nine canteens it was 20–30 clients, and in 11 canteens 31–50 clients. One staff member served over 51 consumers in four canteens. This is an important aspect of the functioning of each canteen, because in a place where a high rotation of customers is observed, there is need to organize the staff well, in order to service many consumers in a short time.

In terms of the attractiveness of the evaluated establishments, in most of them, the interior design was suitable for the consumption of the meal (vases with flowers, tablecloths on the tables, decorations: mirrors, paintings, properly selected lighting with favorable colors for consumed foods). There was a constant availability of sugar and spices, as well as disposable napkins. 

In some places (one, two canteens), irregularities were found in this regard. In nine canteens, there were reservations about the sound system, which was not suitable for the place, disrupted customer conversations which created an inappropriate atmosphere in the dining areas. It is interesting to note that in all of the studied places, specializing in nutrition of students and university staff, there were no promotional campaigns for clients: discounts or rebate vouchers, loyalty cards.

Meals offered in canteens. In all rated canteens (*n* = 25) satisfactory marks were obtained in terms of the variety of offers for customers (meals and beverages), including vegetarian menu, menu information (on boards), except for nutrition information (Table 3). The best inspection results were obtained when assessing meals. This means paying special attention to the nutritional value, variety of meals and their quality. This is a positive aspect in the context of promoting proper dietary patterns. The meals offered varied in culinary techniques as well as offers. Snacks (sandwiches, salads), at least two types of soups (e.g., krupnik, tomato soup, broth, vegetable cream soup) and vegetarian dishes were offered. The choice of main courses was quite large (e.g., pancakes, typical Polish dishes: dumplings, potato dumplings, Silesian dumplings, as well as stews, a dish consisting of meat, starch and salad, pasta with toppings), including fast food dishes (hamburgers, hot dogs, French fries, tortillas etc.). The following was available for dessert e.g. buns, yeast dough, fruit pies, yogurt. Information on allergens in the menu was available only in oral form. In terms of health-promoting menu (dishes with reduced energy value, with a lower content of fat, sodium, sucrose), their presence on the menu was declared in 21 establishments. In four establishments, there was no such offer at all, but while the choice was at a satisfactory level for the remaining ones. 

In terms of food storage after preparation, in bain-maries or in refrigeration conditions, a satisfactory level was also found. Only the sandwiches were stored under cold storage but should be prepared on a regular basis. It should be emphasized, that bread under the cold conditions becomes stale, and therefore, for the quality of sandwiches, it would be better not to store them under refrigeration conditions. Probably in the evaluated canteens, due to the small number of staffs, it is not possible to prepare sandwiches to order of customer. 

In summary, the total the inspection of 25 canteens showed that one canteen represented a fairly low level of quality catering services rated only at 12 points (48% of max. points)), but as many as 20, obtained only up to 14–19 points (56–75% of max. points), but only four canteens were rated 20–21 points (80–84% of max. points). None of the establishments received a maximum grade (25 pts). This may indicate that the owners of the canteens are not paying too much attention to the quality of catering services, including hygiene, customer service and food aspects. 

The opinions of the respondents participating in our study differed significantly from the results obtained in the inspection. The correlation between these evaluations were not found. It can be assumed that the level of knowledge of the respondents in the field of consumer service as well as hygiene and food safety is insufficient, or they do not pay attention to these aspects using the services of university canteens.

## 4. Discussion

### 4.1. Customer Behavior and their Opinion about University Canteens

Our research indicates that only 35.5% of students and staff (*n* = 1250) of the rated universities regularly use canteens, i.e., daily or at least once a week. About 40% of respondents use canteens sporadically. Probably in these group are consumers who have been in university for hours but do not eat a meal at all and eat meals later at home, some of them eat homemade sandwiches, and some of the respondents eat outside of the university, especially students and staffs whose buildings are located in the central city. Rational, regular nutrition is very important for maintaining good health. It has been shown that people who use canteens more often than others, choose full set dishes, i.e., soup and main course, or incomplete main course. In contrast, people who use canteens less often, rather come to canteen to buy drinks and to quench their thirst, as well as to buy a dessert and satisfy the “whim”. 

It should be emphasized that traditions of eating out are just forming in Poland. The number of catering establishments has been growing since 1989, including the number of bars and canteens, which in 2017 amounted to 19,410 (27.7%) and 4221 (6%) respectively [48], and this group of catering establishments includes establishments located at universities. Staff and students usually take their sandwiches from home. Hence, quite numerous respondents declared buying drinks (45.5%), because they go to the canteen to eat their own meal and buy a drink. The results of our study indicate the need to educate students in the field of systematic nutrition as a prerequisite for preventing of diet related diseases.

It is interesting to note that the canteen consumers quite often choose to buy soups (39.2%) and sandwiches (28%). These are meals that can be eaten relatively quickly. It should be added here, that there is no official lunch break in Poland, and the breaks between classes are quite short, which does not allow too long waiting times for a meal in canteen. Moreover, eating on the run has been associated with higher intakes of soft drinks, fast food, total fat and saturated fat intake, but a lower intake of more healthy foods such as fruit and vegetables [49]. This can be a postulate for people involved in planning student classes in determining the time of lunch. At the same time, it is important to create a specific habit of eating a full and nutritious meal, not only snacks. 

It was shown that the frequency of using canteens and menu selection depended on the financial situation of the respondents. This is in agreements with reports from previous research [50]. Students did not agree to the prices set by the canteen management, for these were too high and not affordable. The economic aspect is crucial in the selection of foods by the student [50]. According to some authors [28], a less expensive alternative might be skipping lunch and/or eating sandwiches or snacks. Research by other authors [37] stated that the canteen is not a good place for students to stay and dine in. A significant group of respondents (24.5%) in this study ordered fast foods such as hamburgers, hot dogs, French fries, and tortillas. Other authors [28,43] also indicated, that students have unhealthy eating behaviors, including high intake of fast foods, snacks, sweets, soft drinks, and low intake of fruits, vegetables, fish, whole grains and legumes [43]. 

No nutritional programs were used in the evaluated canteens, as was the case in some European countries [51]. It is known that the environment can exert a strong influence on people’s food decisions, e.g., nutrition information in menu [51]. In order to facilitate students to make more healthy food choices and to develop healthy eating habits, it is important that the food environment is healthy. 

The respondents rated three parameters of the canteen: meals, hygiene and customer service. It can be seen here that people who used full set (main course, or incomplete main course) were more restrained in assessing the quality of services. In contrast, people who usually bought beverages and desserts, more often rated services as positive. This may be due to the short stay in canteen, a visit due to social reasons, which of course affects the better perception of quality by respondents. 

Similar assessments of the quality of services in the university canteen were obtained in other studies [52]. Most of students were satisfied with the quantity of food and ranked the quality of food as “medium”. In other studies [29,53], it was determined that the university students found the quality of food to be insufficient and this situation was effective in the low levels of satisfaction from services. Food quality aspects such as careful handling, cleanliness while serving customers, quality and menu variation, as well as price fairness were considered important by university students dining at the cafeteria [20].

### 4.2. Quality of Canteen’s Services Estimated by Inspections

Our research has shown that respondent ratings do not fully coincide with canteens/cafeterias assessments obtained during inspections. This may indicate that consumers’ attention is not paid to the quality of services or they have a lack of knowledge about the proper functioning of gastronomy. The most discrepancies were observed in the assessment of hygiene in the dining area and personnel hygiene. 

Inspection of canteens showed unsatisfactory level of service in all aspects assessed. Low overall ratings, in terms of hygiene, as well as in consumer service indicate that there is a lot to be done regarding quality at university canteens. Meals were rated the highest - 6 points (median, max. 12). The most incompliances were in the field of personnel hygiene, such as use of disposable gloves, separation of money from food, use of proper working clothes when preparing meals. Similar results were obtained by other authors [52]. Students who use University canteens reported some problems regarding hygienic condition inside and outside the dining services and have stated that more attention should be paid to personal health of students and staff by the appropriate authorities of the University.

There were fewer irregularities in consumer service. In most canteens, no greeting or farewell was used, while speed of service and time spent waiting for order was on satisfactory level in most of the canteens. Attractiveness of canteens, music, and availability of spices, sugar and disposable napkins was estimated as rather satisfactory. Other authors [21,22] observed that, the atmosphere of establishments was found to be effective in satisfying consumers on services. According to Chang et al. [20] the spatial arrangement of seating, quality of interior design, and suitability of background music do not influence students’ satisfaction level with the service quality of the University cafeteria operators. In contrast, Saglik et al. [54] concluded that hygiene dimension had a higher impact on customer satisfaction than the service quality dimension, also that the atmosphere did not have a significant effect on satisfaction. 

In evaluated canteens, promotion and discounts for students were not used. Incentives for constant use of canteens, through applications, loyalty cards, discounts for regular customers are a way to attract customers. None of these forms were used in the evaluated canteens. None were also presented inside the menu, i.e., offers like a soup of the day at a promotional price. The lack of such activities may result from the lack of competition on the one hand, and on the other hand from the demand that remains stable and results from the number of students participating in the classes. The practice is to have only one canteen in the buildings of Warsaw Universities. The occurrence of two catering establishments offering a wide range of food is rare. 

The best inspection results were obtained when assessing meals. They were rated as satisfactory or very good in terms of variety of meals and beverages, and proper storage of meals. In all canteens, there was a board with information about the menu and prices, vegetarian menu, while there was no nutritional information about energy value, and even allergens information, which could only be obtained orally, and which is legally required [55]. Four facilities did not have healthy foods menu. A health-oriented offer with reduced energy, salt and sucrose content is currently important for many consumers who pay attention to their dietary choices. 

In Polish catering establishments, the main attention was usually focused on dietary aspects of the production, whereas the technological and hygienic aspects, including the safety and quality of food production, were not treated as a priority [9]. Results of other authors [56] indicated that the food service hygiene is one of the top three considerations when consumers select a dinning place. An attractive menu, with different food varieties such as vegetarian option, international dishes, at a reasonable price should be offered to the customers and served in an appropriate ambience that can energize their enthusiasm for dining at the university cafeteria [1]. 

## 5. Conclusions

The obtained results have an interesting cognitive aspect. The students’ view can be used by the authorities to improve the quality and quantity of food and hygienic condition of food services in universities. Analysis of consumer responses can provide knowledge about their behaviors in canteens, which are important for the entrepreneur (canteen organizer), for authorities, and for those who analyze behavior in an effort to determine their impact on health, fitness, etc. 

We suggest the need for an evaluation guide for university canteens in Poland. This could help owners improve the quality of service in canteens. The evaluation of the quality of nutrition in canteens should be continued due to the prevention of diet-related diseases. This group of canteens is usually overlooked in research. However, it is very important to evaluate the quality of service taking account not only quality of meals but other aspects of catering services too. Customers of University canteens are usually young adults, whose eating behavior is still shaped. The right quality of meals, eating in a good atmosphere and maintaining proper nutrition and hygiene standards are extremely important. It is also important to raise consumer awareness in this area. 

The respondents’ assessments did not coincide fully with the canteen’s assessments obtained during the inspection, which indicates that consumers do not pay attention to the quality of services in student canteens, or they have low knowledge of the proper conduct of staff, including hygiene of food production and distribution. Respondents rated canteens in three aspects: meals, hygiene in canteens and customer service. People who ate a full set of meal were more critical of the quality of catering service than people who ordered a dessert or drink. 

The best inspection results were obtained when evaluating meals. This means that the owners of canteens, which are run by private owners in Poland, are trying to attract customers, and paying special attention to the nutritional value, variety of meals and their quality. However, canteens have must also comply hygiene requirements, which not fully was met which was stated during inspection. 

To meet the customer’s expectations, it is essential that food service operations deliver not only a quality product, but quality service as well. Further research is needed to confirm these results in the overall student and academic population in Poland and to understand the determinants of university canteen utilization. These research will also be important (from a nutritional point of view) for improving the nutritional quality of students and academic staff. 

## Figures and Tables

**Table 1:** Questionnaire structure.

Question		Variants of Answers
1	**The frequency of visit university canteen:** sporadically, once a month, 2 or 3 times a month, once a week, daily, never	Choose the right answer
2	**The reasons for visiting to the canteens: ** food intake, rest between classes, social meeting, preparation for classes, others	Choose the right answer
3	**Type of meals consumed in canteen**	soups; main course full set (e.g.,: meat, potatoes, salad), main course incomplete set (meat + salad or only meat); fast food meals; sandwiches, salads, desserts, fruits, beverages
4	**Frequency of dish consume: ** soups, main course, fast food, sandwiches, salads, desserts, fruits, beverages	(1)—daily, (2)—2 or 3 times a week, (3)—once a week, (4)—2 or 3times a month, (5)—once a month, (6)—sporadically, (7)—never
5	**Evaluation of dining area:** interior design, attractiveness of equipment, atmosphere of establishment, music – variety and volume, table cleanliness, floor cleanliness, sugar availability, availability of spices, availability of disposable napkins **Evaluation of customer service:** speed of service, order processing time, service time - queuing, staff professionalism, staff politeness, staff outfit **Evaluation of canteens’ menu** **quality of soups:** taste, smell, the way of serving; quality of main course: taste, smell, consistency, the way of serving; variety of meals, availability of healthy meals, prices of soups and main courses	Scale: very bad (−2); bad (−1); no opinion (0); satisfactory (1), good (2), very good (3)
6	**Sociodemographic data: ** Gender: women, men; Age: 19–25 years old, above 26 years old; Education: secondary school, higher education; Dwelling place: city over 500,000 inhabitants, city up to 100,000 inhabitants, village; Evaluation of financial status: very good, good, bad, no answers	Choose the right answer

**Table 2:** Characteristics of the surveyed sample of respondents.

Population Features	Group	Number of Respondents	Percentage of Respondents
Total	--	1250	100.00
Gender	Women men	776 474	62.20 37.80
Age	19–25 years old over 26 years old	1101 149	88.08 11.92
Education	secondary school higher education (university)	1064 186	85.12 14.88
Dwelling place	big city (city over 500,000 inhabitants) small city (city up to 100,000 inhabitants) village	544 455 251	43.52 36.40 20.08
Financial status in own opinion	very good (high income—3) good (average income—2) bad (low income—1) no answer (Income—?)	274 426 467 83	21.92 34.08 37.36 6.64

**Table 3:** Results of inspection (evaluation of canteens) (*n* = 25).

Specification	Evaluated Aspects and Parameters		Number of Canteens Evaluation		Average	Median (Min-Max)
			0 *	1		
**Hygiene aspects** **(max. 9 pts)**	Hygiene of dining area and tableware	cleanliness of tables cleanliness of floor cleanliness of tableware (dishes, cutlery)	5 6 0	20 19 25	5.08	5 (3–7)
	Personal hygiene of staff	working clothes the use of headgear neatness of clothing use of disposable gloves separation money from food hygiene handling with food	14 14 9 25 25 0	11 11 16 0 0 25		
**Customer service aspects** **(max. 10 pts)**	Customer service	welcome customers farewell customers speed of service order processing time	20 22 8 6	5 3 17 19	4.40	4 (3–5)
	Attractiveness of canteens	interior design, attractiveness music (variety, volume) sugar availability availability of spices availability of disposable napkins promotion - discount	2 9 2 2 1 25	23 16 23 23 24 0		
**Food aspects** **(max. 6 pts)**	Meals	variety of meals and beverages information in menu, including nutritional information vegetarian menu healthy foods menu the way of meals storage allergens information	0 0 25 0 4 0 0	25 25 0 25 21 25 25	5.84	6 (5–6)
Total (max. 25 pts)					17.04	17 (12–21)

* Scale: 0-unsatisfactory, 1-satisfactory.
